# Supplementary material for: Patterns of Intron Gain and Loss in Fungi
Source: PLoS Biol. 2004 Nov 30;2(12):e422. doi: 10.1371/journal.pbio.0020422 (PMC532390; doi:10.1371/journal.pbio.0020422)
Supplement: Table S1 — Also available at http://genes.mit.edu/NielsenEtAl/. (4.3 MB ZIP). [file pbio.0020422.st001.zip › NielsenEtAl/html/481.html]

AN0257.1.NCU00316.1.MG06332.1.FG05379.1


```
 CLUSTAL W (1.82) Multiple Sequence Alignments - Introns Inserted


Sequence 1: MG06332.1	348 aa
Sequence 2: FG05379.1	340 aa
Sequence 3: AN0257.1	336 aa
Sequence 4: NCU00316.1	339 aa
Alignment Length: 351 aa
Number Identitical Residues: 178 aa
Alignment Score (without introns) 8505


MG06332.1 	MSAQAKSAPIGPWARATAGATGAVLANALVYPLDI2VKTRLQVQVKPKTGET---PAPGS
NCU00316.1	MAAHSKPAQLGPWGKATAGAAGAAFANVLVYPLDL2IKTKLQVQVKKSADVEKQEATASN
FG05379.1 	MAAQSKPE-ISPWGRAVAGATGAVLANALVYPLDI2VKTRLQVQIKPDPSKE---PSPTD
AN0257.1  	MAGQSKPA-LSPWGSAVAGATGAVLANAIVYPLDL2VKTKLQVQVKKAGEAE---SKDSD
          	*:.::*.  :.**. *.***:**.:**.:*****: :**:****:*         .   .

MG06332.1 	EPHYNSTWDAITKIMEQDGLGGLYAGMGGSLIGVASTNFAYFYWYSVVRSLYFKSVSKTG
NCU00316.1	EVHYKGTWDALTKIKNAEGIAGLYAGMNGALLGVTSSNFAYFYWYSIVRTLYLQYQ-KS-
FG05379.1 	EPHYTSTWDAISRIVADDGIKGLYAGMNGSLIGVASTNFAYFYWYTIVRTLYLKSR-KT-
AN0257.1  	DVHYKSTWDAITKIMDKEGVEGLYSGMAGSLIGVASTNFAYFYWYSVVRTLYMASE-RV-
          	: **..****:::*   :*: ***:** *:*:**:*:********::**:**:    :  

MG06332.1 	IAPSAPSTAVELSLGAIAGAVAQLCTIPVAVVTTRQQTQSKEERKGLLDTAKDVIESEDG
NCU00316.1	--DAHPSTAAELSLGAVAGALGQLFTIPVAVVTTRQQTQSKEDRKGIIDTAREVVEGEDG
FG05379.1 	--DANPSTVVELGLGAIAGAIAQVFTIPVAVVTTRQQTASKCDRKGLMATAREVIDGPDG
AN0257.1  	--PKPPGTAIELSLGAVAGAVAQIFTIPVAVITTRQQTQPKDDKKGLIETGREVVNSEDG
          	     *.*. **.***:***:.*: ******:****** .* ::**:: *.::*::. **

MG06332.1 	VFGLWRGLKASLVLVVNPAITYGAYERLKVLFFPGKSNLKPWEAFV1LGAMSKSLATIAT
NCU00316.1	ITGLWRGLKASLVLVVNPAITYGAYERLKDILFPGKNTLKPWEAFL1LGALSKSIATIVT
FG05379.1 	VSGLWRGLKASLVLVVNPAITYGAYERLKEVMYPGKTSLRPGEAFL1LGAMSKALATIAT
AN0257.1  	WSGLWRGLKASLILVVNPAITYGAYQRLKDIIFPGKKSLKPWEAFL1LGALSKAMATIAT
          	  **********:************:*** :::***..*:* ***: ***:**::***.*

MG06332.1 	QPLIVAKVGLQSKPPASRKGKPFKSFVEVMRFIIDNDGPTGLFKGIGPQILKGLLVQGFL
NCU00316.1	QPLIVAKVGLQSKPPAARNGKPFKSFVEVMEFIVKNEGALSLFKGIGPQLLKGFLVQGIL
FG05379.1 	QPLIVAKVGLQSKPPPARNGKPFTSFVEVMKFIIEHEGMLGLFKGIGPQILKGLLVQGIL
AN0257.1  	QPLIVAKVGLQSRPPPARNGKPFKTFSEVMRFIVENEGPLSLFKGIGPQILKGLLVQGLL
          	************:**.:*:****.:* ***.**:.::*  .********:***:****:*

MG06332.1 	MMTKER2VELLFILFLRYIQTARSKQLYSAADLASKATSAASGAASKVSPLVTK
NCU00316.1	MMTKER2VELMFILFMRFLSKLRSQQLGKAAALAAAAKVAS-PVIAK-------
FG05379.1 	MTAKER2VELMFILLIRYIKSIQLRKISRAAEKTAVAAAAHSVAAQPVTST---
AN0257.1  	MMTKER2MELLFIVLFAYLKGLREKKLRKAVDAAASKAQASLPATLK-------
          	* :*** :**:**::: ::.  : :::  *.  ::    *   .
```
